# Inhibition of liver cholesterol synthesis by a diet-induced gut hormone

**DOI:** 10.1016/j.apsb.2024.07.026

**Published:** 2024-08-05

**Authors:** Xiabing Huang, Jianping Ye

**Affiliations:** a Metabolic Disease Research Center, Zhengzhou Central Hospital Affiliated to Zhengzhou University, Zhengzhou 450007, China; b Tianjian Laboratory of Advanced Biomedical Sciences, Academy of Medical Sciences, Zhengzhou University, Zhengzhou 450052, China

**Keywords:** Cholesterol, Gut-derived hormone, Cholesin, Intestine, Diet

The liver controls blood cholesterol levels through biosynthesis and recycling of cholesterol^1^. It is a major target for pharmacological interventions of blood cholesterol in the clinical settings, where Statins inhibit cholesterol biosynthesis, and PCSK9 inhibitors enhance recycling of low-density lipoprotein (LDL) from blood into the liver^2^. A recent Cell paper reports that the liver activity is regulated by a diet-induced gut hormone, Cholesin, in the gut–liver axis for control of cholesterol biosynthesis^3^.

Cholesterol has well-established activities in the maintenance of cell membrane structure, biosynthesis of bile acids and steroid hormones in the physiological conditions^4^. Over supply of cholesterol increases the risk for cardiovascular diseases, neurodegenerative disorders, and hormone disorders through hypercholesterolemia in the pathological conditions^5^^,^^6^. Cholesterol is required by neuronal cells for various functions of the brain, and a lack of cholesterol supply impairs the neuronal cell functions leading to early neuron degeneration for aging diseases including Alzheimer's disease^7^. Consequently, modulation of cholesterol levels in the brain may present a novel therapeutic approach for late-onset Alzheimer's disease (LOAD)^7^.

Cholesterol comes from two primary sources in the body: *de novo* synthesis and dietary intake. The synthesis is controlled by the key enzymes, HMG-CoA reductase 1 (HMGCR1) and squalene monooxygenase, which convert acetyl-CoA into cholesterol^8^. Cholesterol together with triglycerides is used to synthesize very low-density lipoproteins (VLDLs) for subsequent release into the bloodstream to supply peripheral tissues. Dietary cholesterol is absorbed by the small intestine and transported to the liver via the portal vein^9^. Blood cholesterol is regulated mainly by LDL derived from VLDL after triglyceride depletion. On the other hand, the liver and other tissues facilitate the clearance of blood LDL through LDL receptor (LDLR)-mediated internalization and degradation of LDL. Additionally, high-density lipoprotein (HDL) helps transport excess cholesterol from tissues to the liver. Cholesterol is stored in cells as cholesterol esters and re-esterified by the enzyme acyl-coenzyme A:cholesterol *O*-acyltransferase (ACAT) for future use^10^. Cholesterol plays a crucial role in maintaining immune cell function although in certain circumstances it may have inhibitory effects on immune cells. Within the tumor microenvironment, tumor-associated macrophages have been found to catalyze the oxidation of cholesterol leading to the production of 7*α*,25-hydroxycholesterol oxidation products that suppress macrophage function and promote tumor progression^11^.

The communication between the liver and intestine is facilitated by the gut–liver axis, which include various gut hormones such as somatostatin, cholecystokinin, gastrin, glucagon-like peptide-1, glucagon-like peptide-2, gastric inhibitory peptide, peptide YY, neurotensin, and fibroblast growth factor. Those hormones play a crucial role in regulating liver functions as well as other organs, including the gastrointestinal tract, pancreas, gallbladder, and nervous system^12^. However, it is important to note that these hormones do not directly regulate liver cholesterol biosynthesis. The novel gut hormone, Cholesin, has been identified for this unique role recently^3^.

Cholesterol synthesis in the liver is impacted by the availability of dietary cholesterol. Absorption of dietary cholesterol occurs in the intestine through vesicular endocytosis mediated by Niemann–Pick C1-like 1 (NPC1L1). Hepatic NPC1L1 facilitates the conversion of cholesterol into bile acids, thus contributing to overall cholesterol homeostasis in the body^13^. The gut microbiota also affects cholesterol metabolism through the production of enzymes^14^^,^^15^. Nevertheless, the factors and mechanisms responsible for the regulation of NPC1L1 remain largely unidentified. Lu et al.^16^ revealed that high-sugar and high-fat diets increase the phosphorylation of ubiquitin-specific peptidase 20 (USP20), stabilizing HMGCR1 and promoting liver cholesterol synthesis. Inhibition of USP20 accelerates HMGCR1 degradation, reducing blood and liver lipids. However, hormone's role in the dietary effect remains unknown. Hu et al.^3^ examined regulatory mechanisms controlling intestinal cholesterol absorption and hepatic cholesterol synthesis, identifying the hormone Cholesin as an inhibitor of both processes. This novel hormone exhibits distinct activity from other gut hormones.

In the new study^3^, mice were administered a high-cholesterol Western diet following an overnight fast to elevate their dietary cholesterol intake. Subsequently, protein enrichment and silver staining analyses were conducted on the mouse plasma samples, which indicated a notable augmentation in bands approximately 23 kDa in size. The diet facilitated the secretion of a homologous protein within the intestine, which interacted with the cell surface receptor GPR146 to suppress the cAMP–PKA–ERK pathway, thereby downregulating SREBP expression and ultimately inhibiting cholesterol synthesis. That homologous protein was found to be encoded by the uncharacterized gene 3110082I17Rik, a protein of human C7orf50. As a result of its suppressive impact on hepatic cholesterol synthesis, the compound was designated “Cholesin” by researchers. By employing a mouse model with intestine-specific Cholesin knockout, it was elucidated that Cholesin functions as an intestine-specific hormone in response to cholesterol stimulation. Cholesin is predominantly expressed in absorptive intestinal cells, with its secretion contingent upon cholesterol absorption facilitated by NPC1L1. Analysis of genome-wide association study (GWAS) data indicated a significant association between the single nucleotide polymorphism (SNP) rs1007765 in Cholesin and total cholesterol levels in human plasma. Clinical tests confirmed the negative correlation between Cholesin and plasma total cholesterol levels and LDL-C levels. rs1007765 is located in the enhancer region that facilitates the transcriptional activation of the Cholesin gene. Through experimentation involving mice with Cholesin deficiency specifically in the intestines, the researchers observed that Cholesin interacts with GPR146 to inhibit the PKA signaling pathway, consequently suppressing hepatic cholesterol synthesis mediated by SREBP2. Administration of exogenous Cholesin effectively reduced hepatic cholesterol synthesis in mice and exhibited therapeutic benefits in mouse models of hypercholesterolemia and atherosclerosis, particularly when combined with rosuvastatin. GPR146 is recognized for its role in the regulation of lipid metabolism. In late 2019, Yu et al.^17^ demonstrated that GPR146 is responsive to dietary stimulation, leading to the activation of ERK, upregulation of the SREBP pathway, and promotion of very low-density lipoprotein (VLDL) secretion. In early 2020, Han et al.^18^ further identified the non-coding region mutation rs1997243 in the 7p22 region of the genome as a pathogenic mutation causing hypercholesterolemia through increased transcription of GPR146. Previous research suggested the existence of endogenous agonists of GPR146 in serum, yet the identity of its endogenous ligand remained unknown until the discovery of Cholesin as an inhibitory ligand for GPR146. Research on Cholesin suggests that the regulation of cholesterol metabolism by GPR146 is independent of low-density lipoprotein receptors (LDLRs). Targeting Cholesin or GPR146 with drugs may offer distinct advantages in the management of hypercholesterolemia.

Currently, management of blood cholesterol is dependent on inhibiting cholesterol synthesis and increasing LDL uptake. Statins are commonly used to reduce blood cholesterol to lower the risk of cardiovascular diseases, but they have some adverse effects and limited efficacy^19^. The PCSK9 inhibitors reduce the blood cholesterol levels by promoting LDL re-cycling, although concerns have been raised regarding the cost of this treatment^20^. ATP citrate lyase inhibitors, such as Bempedoic acid, present an alternative method for lowering cholesterol levels; however, their effects on cardiovascular events are not yet fully understood, and they may lead to adverse reactions, such as gout and cholelithiasis^21^. Therefore, there is a need for new strategies that are both highly effective and cost-efficient in reducing blood cholesterol levels. Cholesin, as a potential drug candidate, may offer a novel approach to drug discovery in this area. It is believed that more insights into the physiological and pathological functions of Cholesin are desired (Fig. 1). The study by Hu et al. has some limitations, such as the long-term effects of Cholesin on cholesterol metabolism and overall health are unknown. Additionally, the exact mechanisms by which Cholesin regulates cholesterol synthesis in humans needs to be investigated. In the gut–live axis, future research should focus on understanding how cholesterol biosynthesis in the liver reciprocally regulates cholesterol absorption in the gut, and why increased cholesterol absorption in the gut could not reduce hepatic cholesterol biosynthesis. Genetic variations, hormonal regulation, dietary influences, interactions with gut microbiota, and pathophysiological conditions such as obesity or insulin resistance may all contribute to this complex interplay. These questions are crucial for a comprehensive understanding of the gut–liver axis in the body.

**Figure 1:**
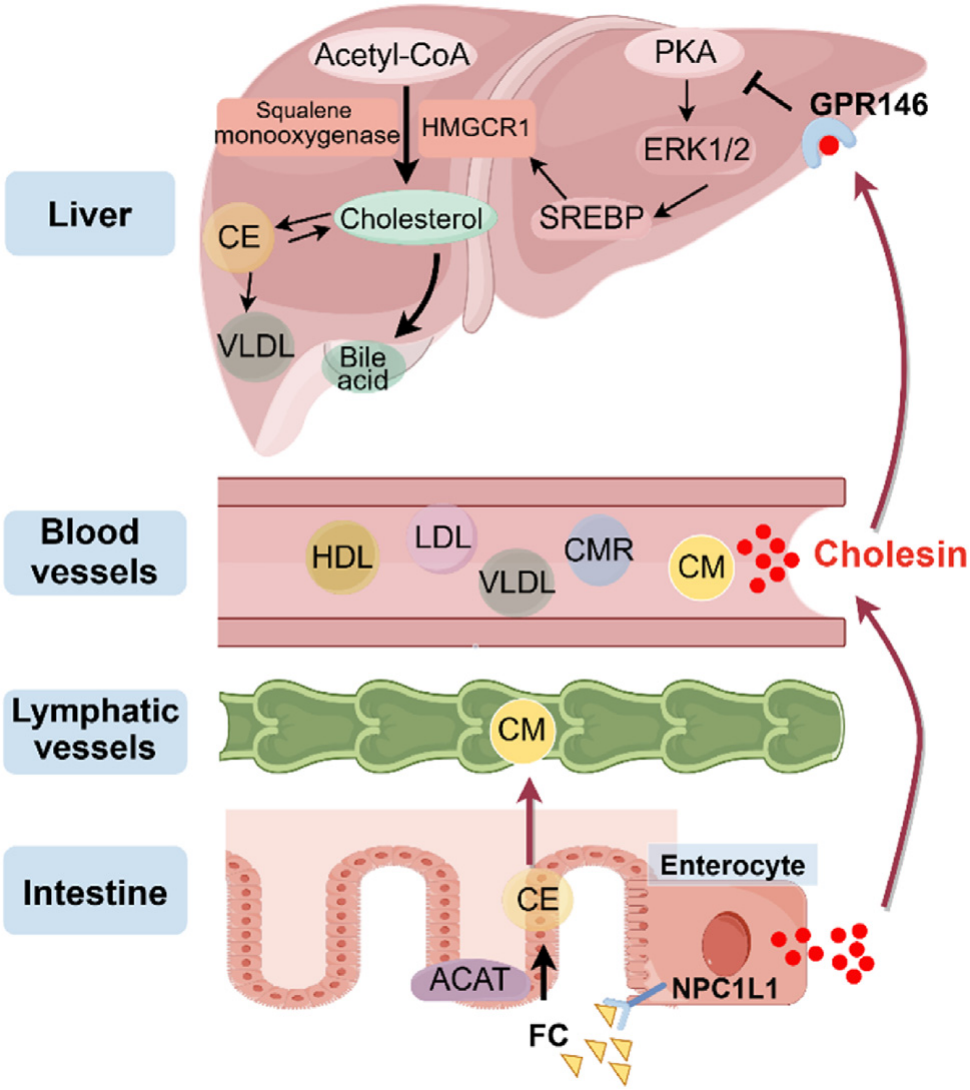
Cholesin in regulation of cholesterol metabolism (By Figdraw). FC: free cholesterol; CE: cholesterol ester; CM: Chylomicron; CMR: Chylomicron remnant.

## Author contributions

Xiabing Huang: Writing – original draft. Jianping Ye: Writing – review & editing, Validation, Project administration, Funding acquisition.

## Conflicts of interest

The authors declare no conflict of interest.
